# Coagulation abnormalities in SARS-CoV-2 infection: overexpression tissue factor

**DOI:** 10.1186/s12959-020-00250-x

**Published:** 2020-12-15

**Authors:** Zahra Eslamifar, Mahin Behzadifard, Masoud Soleimani, Saba Behzadifard

**Affiliations:** 1 Dezful university of medical sciences, Dezful, Iran; 2 Faculty of Medical Sciences, Tarbiat Modares University, Tehran, Iran; 3 Department of Anatomical Sciences, Faculty of Medical Sciences, Tarbiat Modares University, Tehran, Iran

**Keywords:** Coagulopathy, SARS-CoV-2, COVID-19, Tissue factor, Thrombosis, Angiotensin converting enzyme, Angiotensin II

## Abstract

Among the pathways and mediators that may be dysregulated in COVID-19 infection, there are proinflammatory cytokines, lymphocyte apoptosis, and the coagulation cascade. Venous and arterial thromboembolisms also are frequent in COVID-19 patients with the increased risk of some life-threatening complications such as pulmonary embolism, myocardial infarction, and ischemic stroke. In this regard, overproduction of proinflammatory cytokines such as IL-6, IL-1β, and TNF-α induce cytokine storms, increase the risk of clot formation, platelet activation, and multiorgan failure that may eventually lead to death among these patients. Surface S protein of SARS-CoV-2 binds to its target transmembrane receptor, named as angiotensin converting enzyme 2 (ACE2(, on various cells such as lymphocyte, alveolar cells, monocytes/macrophages, and platelets. Notably, the activation of the coagulation cascade occurs through tissue factor (TF)/FVIIa-initiated hemostasis. Accordingly, TF plays the major role in the activation of coagulation system during viral infection. In viral infections, the related coagulopathy multiple factors such as inflammatory cytokines and viral specific TLRs are involved, which consequently induce TF expression aberrantly. SARS-COV-2 may directly infect monocytes/ macrophages. In addition, TF expression/release from these cells may play a critical role in the development of COVID-19 coagulopathy. In this regard, the use of TF- VIIa complex inhibitor may reduce the cytokine storm and mortality among COVID-19 patients.

## Introduction

Although the mechanisms that activate coagulation cascade in SARS-CoV-2 infection are still unknown, they are evident to be linked to inflammatory responses [1].

inflammatory response of COVID-19 infection may be self-limited in those patients experiencing mild symptoms; however, in a smaller fraction of patients with COVID-19 infection, it is associated with the inductions of coagulopathy, disseminated intravascular coagulation (DIC) [2], cerebrovascular accidents [3], pulmonary thromboembolism, and multiorgan failure [4]. COVID-19 coagulopathy was also indicated to be associated with an increase in procoagulant factors like fibrinogen as well as a strong increase of D-dimers that are linked with a higher mortality rate [5, 6] .

Enhancement of D-dimer above 1000 ng/ml is known as a risk factor of death in COVID-19 patients [5]. Moreover, SARS-CoV-2 infected cells down-regulate the expression of ACE2 protein. As a result, the accumulation of angiotensin II (AT-II) occurs secondary to the reduced ACE2 in COVID-19 infection. In addition, the increased AT-II may promote clot formation via having interactions with platelets and endothelial cells [7]. In promoting microvascular damage associated with AT-II, IL-6 and TNF-α may play direct roles. Correspondingly, AT-II and TNF-α have been implicated in promoting the overexpression or release of TF in platelets and macrophages [8]. Additionally, promoting an increase in TF due to antiphospholipid antibodies, may also be associated with COVID-19 coagulopathies [9]. Therefore, TF may play a role in coagulation cascade irreregulations in COVID-19 patients. Besides, some additional pro thrombotic events observed in COVID-19 patients, including an increase in serum IL-6 that has potentially characterized activities platelets [10] and coagulation factors [11], T lymphocytes [12] as well as IL-6 and TNF-α may play direct roles in promoting micro vascular damage associated with AT-II [13].

Tissue factor (TF) is the physiological activator of extrinsic coagulation pathway playing the central role in hemostatic protection of vital organs such as lung, brain, and heart [14].

TF is a transmembrane glycoprotein that is mostly restricted to the cells of the sub endothelial vessel wall under physiologic conditions (i.e., pericytes, smooth muscle cells and fibroblasts), which are not in direct contact with the blood [15].

In healthy individuals, by the exclusion of 1–2% of blood circulating monocytes that expresses intracellular TF in a low levels, blood leukocytes cannot express it. It is noteworthy that blood is not thought to contain functional TF because of its high procoagulant activity [16].

After the disruption of the vessel wall or after the upregulation of TF on monocytes under inflammatory situation transmembrane, TF would have a contact to FVII and FVIIa with a high-affinity. Moreover, TF stabilizes the catalytic site of FVIIa on plasma membrane to provide an optimal interaction with FIX and FX, similar to its substrates. The activation of FX and FIX proceeds coagulation protease cascade, which consequently produces fibrin clot.

### TF procoagulant state in viral infections

The activation of coagulation cascade during viral infections may be considered as a protective response and a limit spread of the pathogen [17].

Endosomal Toll Like Receptors (TLRs) detected viral infections mainly are specific for nucleic acids. TLR3 sensing viral double strand RNA (ds RNA), and TLR7 and TLR8 recognize viral single strand RNA (ssRNA) and TLR9 is triggered by viral DNA [18]. The TLR3 agonist polyinosinic polycytidylic acid (poly I:C) induces TF expression in the cultured endothelial cells and finally activates the coagulation cascade in mice [19].

TF appears to be the major activator of the coagulation system during viral infection. For instance, Ebola, Denge, HIV, HSV, Marburg, Hantavirus, and SARS-COV-2 were all shown to be associated with coagulation activation and thrombosis [20–26].

In addition, respiratory viruses such as influenza A, influenza B, parainfluenza-1, respiratory syncytial virus, adenovirus, and cytomegalovirus induce infection in human endothelial cells during culturing. Accordingly, these viruses induce TF expression in various cells, including monocytes and ECs that are associated with the activation of coagulation and thrombosis. Moreover, the pronounced activation of coagulation in elderly patients with an influenza virus infection was previously described [27].

TF-dependent activation of the coagulation cascade in HSV1 infection of both of the cultured endothelial cells and in mice leads to the increased infection [28].

TF-VIIa complex inhibitors may also reduce the cytokine storm and mortality in COVID-19 patients [29].

Under the procoagulant or proinflammatory conditions, both platelets and monocytes have been shown to express TF. TF expression in monocytes and platelets obtained from the HIV infected patients played a major role in HIV-associated coagulopathy.

In severe cases of COVID-19, TF expression was described from the activated monocytes. The induced TF expression in monocytes during COVID-19 were also indicated to be associated with severity and mortality in severe COVID-19 patients [30].

### AT-II accumulation and TF overexpression

SARS-CoV-2 attaches to and enters those cells that express ACE-2 protein. This receptor has been identified on pneumocytes, macrophages, and monocytes, as well as on the cardiomyocytes, trachea, and bronchi [31, 32]. It is noteworthy that SARS-CoV-2 binding induces an immediate down-regulation of ACE-2 transmembrane receptor [33], and as a result, leads to a secondary increase in its endogenous substrate levels, as AT-II. This acute accumulation in levels of AT-II may have direct implications for the vascular endothelial, coagulation, and immune responses [34–36].

In some situations like systemic sepsis, monocytes / macrophages respond with the increased synthesis and release of TF, and these findings have been linked to coagulation cascade dysegulations [37]. Similarly, macrophage activation and the resulting increases in TF production may also occur in the ACE-2 down-regulation and secondary accumulation of AT-II, in order to promote deregulated thrombosiss. Platelets also express TF in response to infectious situation, which can interact with monocytes /macrophages cells [38]. Correspondingly, such findings have been reported in platelet activation response to AT-II-mediator as well [39, 40].

In case of dengue hemorrhagic fever, Wills et al. [41] described that dengue infection may activate fibrinolysis pathway by directly degrading fibrinogen, thus promoting the secondary activation of pro-coagulant state including increases in plasma levels of TF. Geisbert et al. in their study postulated that the expression of TF was only observed in monocytes /macrophages with morphological changes of Ebola virus replication, suggesting that the expression of TF may be directly induced by Ebola infection [24].

Bautista-Vargas have described that the secondary accumulation AT-II in SARS-COV-2 infection may also induce overexpression of TF protein. It is likely that the overproduction of TF can be considered as the primary initiating factor for the observed coagulopathy in COVID-19 patients [8].

## Conclusion

Venous and arterial thromboembolisms are frequent among critically ill COVID-19 patients with the increased risk of some life-threatening complications such as pulmonary embolism, myocardial infarction, and ischemic stroke [42, 43].

Although the mechanisms of hypercoagulopathy in SARS-CoV-2 infection are still unknown, they appear to be associated with inflammatory responses [44].

In this regard, rapid investigation is required to determine which coagulopathy pathways mostly contribute to morbidity and mortality in COVID-19 infection. Among these critical mechanisms, F overexpression may play a critical role. Previous studies have shown that inflammatory cytokines and agonist of viral specific TLRs could induce TF expression. In addition, the presence of ACE-2, as SARS-COV-2 receptor on monocyte/macrophage cells, may directly induce TF overexpression in these cells.

Further studies are required to determine the exact role of TF that might be helpful in describing the pathogeneses of thrombosis and coagulopathies associated with COVID-19 infection. In addition, we suggest that the inhibition of TF-FVIIa complex may reduce the cytokine storm and mortality in COVID-19 patients.

## Data Availability

Not applicable.

## Abbreviations

*SARS-CoV-2*: Severe acute respiratory syndrome coronavirus 2
*COVID-19*: Coronavirus disease
*ACE-2*: Angiotensin converting enzyme-2
*AT-II*: Angiotensin II
*TF*: Tissue factor
*IL-6*: Interleukin 6
TNF-α: Tumor necrosis factor-α
